# Peutz-Jeghers syndrome: revisited

**DOI:** 10.4322/acr.2021.384

**Published:** 2022-05-13

**Authors:** Vitorino Modesto dos Santos, Lister Arruda Modesto dos Santos, Laura Campos Modesto

**Affiliations:** 1 Hospital das Forças Armadas, Department of Medicine, Brasília, DF, Brasil; 2 Universidade Católica de Brasília, Brasília, DF, Brasil; 3 Hospital Francisco Morato de Oliveira, Service of General Surgery, Advanced Program of Oncosurgery and Videolaparoscopy, São Paulo, SP, Brasil; 4 Centro Universitário de Brasília, Medical School, Brasília, DF, Brasil

**Keywords:** Autopsy, Diagnosis, Peutz-Jeghers syndrome, Polyps

To the Editor

In 1986, Jonathan Hutchinson first described manifestations of Peutz-Jeghers syndrome (PJS). However, the genetic heritance was reported by Jan Peutz (1886-1957) in 1921, and the dominant autosomal heritage was defined by Harold Joseph Jeghers (1904-1990), Victor Almon Mckusick (1921-2008), and Kermit Harry Katz (1914-2003) in 1949.^1^^-^3 Dr. Jan Peutz reported a three-generation study including ten cases of the syndrome; 7 patients had pigmentation in the oral region and multiple small intestine polyps, 2 also had nasal polyps, one had bladder polyps, and jejunum intussusception occurred in one patient.^3^ Jeghers et al.^2^ reviewed the literature about pigmentation on the face, lips, and oral cavity associated with intestinal polyposis and 10 cases were described. They emphasized the oral and perioral melanin deposition and the small intestine polyposis as the main features of PJS, and also established the autosomal dominant inheritance pattern of this syndrome. The eponymous PJS was initially used in 1954, and the first histological description of the hamartomatous polyps was performed by Horrilleno and colleagues in 1957.^4^

This rare autosomal dominant entity is due to a mutation of serine-threonine kinase (*STK11/LKB1*) gene on chromosome 19, and causes mucocutaneous pigmentation, hamartomatous polyps, digestive hemorrhage, intussusception, and malignancies.^1^^-^9 These mutations have been detected in up to 70% of cases in affected families, and in 30% to 67% of sporadic cases.^4^ Approximately 95% of the typical PJS pigmentations like small dark brown, circular or oval macules are distributed around the mouth, eyes, nostrils, and in the extremities.^4^^,^^5^ PJS affects 1/50 000-20 000 people, and is associated with an increased risk of stomach, intestine, pancreas, breast, lung, uterus, ovaries, and testes cancers.^4^^-^7^,^^9^ The incidence of cancers in PJS is almost 15 times higher than in general population.^7^ The lifetime increased risk of malignancy by the age of 70 may be up to 90%.^5^^,^^8^ The lifetime cancer risk of 1644 patients with PJS ranged from 37% to 93%, and the average age of developing any malignancy was 42 years.^7^ The lifetime increased risk of malignancy by the age of 70 years may be up to 90%.^5^ Diagnostic criteria include: (I) two or more PJ polyps; (II) any number of polyps with a family history of PJS; (III) mucocutaneous pigmentations with a family history of PJS; or (IV) one or more PJ polyps and mucocutaneous pigmentations.^5^^-^7 The polyps are pedunculated or sessile, ranging from microadenomas to several centimeters in diameter, may be single or hundreds involving extensive areas.^4^ The histopathologic diagnosis is established with the base on the arborizing pattern observed in the hamartomatous PJ.^4^^,^^6^ Most common PJ gastrointestinal polyps are described in the small bowel (50%), stomach (36%), and colon (21%); extra-intestinal may be in the bladder, nostril, bronchi, or gallbladder.^3^^,^^4^^,^^6^^,^^7^^,^^9^ Gastrointestinal polyps may cause bleeding, intussusception, and even rectal prolapse;^6^^,^^8^ and the intussusception risk by age 10 is 15%, and by 20 years the risk is up to 50%.^7^ Small bowel intussusception is the most urgent and life-threatening of all manifestations.^8^

Differential diagnosis include neurofibromatosis, Cowden and Bannayan-Riley-Ruvalcaba syndromes, multiple endocrine neoplasia, and hereditary mixed polyposis.^4^ Suspected cases of PJS must have genetic testing and informed assent to have children; once an individual is diagnosed with PJS, all their at-risk relatives should be tested.^8^^,^^9^ Patients with PJS must begin screening for polyps at the age of eight, with annual complete physical evaluations and blood exams, besides precocious tumor resections.^4^^-^9 The prevention of surgical complications and monitoring of malignant transformation of the polyps include: control of total blood count, determination of CA-125 and CA-19-9 levels, imaging studies of digestive tract, testes, breast and pelvic organs.^4^^-^9 Consensual guidelines for better managing patients with PJS are lacking. Current recommendations remain poor, needing a multidisciplinary expertise collaboration.^8^^,^^9^ Ideal teams for management and surveillance include gastroenterologists, geneticists, clinicians, surgeons, oncologists, and nurses able to improve the prognosis of the disease.^9^

Recently, the author’s team read the case report by Oliveira et al.^6^ describing the autopsy study of a 32-year-old pregnant woman at about the 30th week of gestation who had nausea, vomiting, and diarrhea for 30 days before being admitted to the emergency room with abdominal colic for 3 days. The physical examination revealed findings consistent with an unidentified acute abdominal condition that caused her death during the first admission evaluation. Worthy of note was a laparotomy that she underwent with 4 years of age, but no information was available on previous personal or familial diagnosis of digestive polyposis. Major autopsy data were arborizing gastrointestinal polyps, mainly in the small bowel. Still numerous gastric and duodenal pedunculated and sessile polyps were found. There was jejunal obstruction by a necrotic polyp, with perforation and peritonitis, and few voluminous sessile colonic masses were detected, but no extra-intestinal polyp.^6^ The authors commented that 95% of intussusceptions develop in the small bowel due to polyps with a diameter greater than >15mm, as disclosed by the postmortem study.^6^ Malignant changes were not detected in the gastrointestinal tract nor in any other organ, besides, no mucocutaneous pigmentation was observed in postmortem examination. The absence of signs of PJS, easily seen by general inspection of the patient, and lack of information about the basic cause of laparotomy made difficult the clinical diagnosis.^6^ In the present context, the authors recommend the additional reading of the article by Scarl et al.,^10^ which is relevant for judicious opinions on the role of necropsy studies in establishing correct diagnosis.

These commentaries on the rare PJS aim to emphasize the role of early diagnosis and attentive multidisciplinary follow-up of the patients since the first decade of life. Moreover, the authors strongly believe that the postmortem study, herein highlighted, contributed to make clear an unsuspected *causa mortis*, favoring the necessary active search for other unsuspected familiar cases of uncommon genetic disorders like PJS.
